# Population genetics and evolutionary history of the wild rice species *Oryza rufipogon* and *O. nivara* in Sri Lanka

**DOI:** 10.1002/ece3.4665

**Published:** 2018-11-11

**Authors:** Salinda Sandamal, Asanka Tennakoon, Qing‐Lin Meng, Buddhi Marambe, Disna Ratnasekera, Arthur Melo, Song Ge

**Affiliations:** ^1^ Department of Agricultural Biology, Faculty of Agriculture University of Ruhuna Matara Sri Lanka; ^2^ State Key Laboratory of Systematic and Evolutionary Botany, Institute of Botany Chinese Academy of Sciences Beijing China; ^3^ Department of Crop Science, Faculty of Agriculture University of Peradeniya Peradeniya Sri Lanka; ^4^ Department of Agriculture, Nutrition and Food Systems University of New Hampshire Durham New Hampshire; ^5^ University of Chinese Academy of Sciences Beijing China

**Keywords:** evolutionary history, *O. nivara*, *O. rufipogon*, population genetics, Sri Lanka

## Abstract

Genetic diversity and population genetic structure of the wild rice species *Oryza rufipogon* and *O. nivara* in Sri Lanka were studied using 33 microsatellite markers. A total of 315 individuals of 11 natural populations collected from the wet, intermediate, and dry zones of the country were used in the study. We found a moderate to high level of genetic diversity at the population level, with the polymorphic loci (*P*) ranging from 60.6% to 100% (average 81.8%) and the expected heterozygosity (*H*_E_) varying from 0.294 to 0.481 (average 0.369). A significant genetic differentiation between species and strong genetic structure within species were also observed. Based on species distribution modeling, we detected the dynamics of the preferred habitats for the two species in Sri Lanka and demonstrated that both *O. rufipogon* and *O. nivara* populations have expanded substantially since the last internal glacial. In addition, we showed that the geographical distribution of the two species corresponded to the climate zones and identified a few of key environmental variables that contribute to the distribution of the two species, implying the potential mechanism for ecological adaptation of these two species in Sri Lanka. These studies provided important insights into the population genetics and evolution of these wild species in Sri Lanka and are of great significance to the in situ conservation and utilization of these wild resources in genetic improvement of rice.

## 1 INTRODUCTION

Knowledge of population genetics and evolutionary history of wild progenitors of cultivated plants is critical not only to understand the evolutionary processes leading to domestication, but also to their conservation and management (Doebley, Gaut, & Smith, 2006; Tanksley & McCouch, 1997). Studies on the level and distribution of genetic diversity in crops and their wild relatives will facilitate the effective utilization of the wild resources in crop genetic improvement and breeding (Tanksley & McCouch, 1997; McCouch et al., 2013). Given the fact that genetic diversity among commercial cultivars decreases substantially in the course of the domestication and breeding, scientists have taken great efforts to explore the genetic variability of wild species to meet the demands for crop breeding. They have particularly focused on the agronomically important traits such as grain yield, grain quality, and sufficient resistance to biotic or abiotic stress, for which genetic variation is limited in the cultivated germplasm (Brar & Khush, 1997; Hajjar & Hodgkin, 2007; Tanksley & McCouch, 1997).

The rice genus consists of diploid and tetraploid species with genome constitutions of AA, BB, CC, EE, FF, BBCC CCDD and HHKK, and HHJJ (Aggarwal, Brar, Nandi, Huang, & Khush, 1999; Ge, Sang, Lu, & Hong, 1999), which is an important gene bank harboring numerous favorable traits and genes that can be used to enhance the genetic basis of rice cultivars (Sang & Ge, 2007; Vaughan, Morishima, & Kadowaki, 2003). In particular, the wild relatives containing AA‐genome have high genetic compatibility with cultivated rice and are the most accessible genetic resources for rice breeding (Sang & Ge, 2007; Vaughan et al., 2003; Zhu & Ge, 2005). Many studies have revealed that various traits derived from wild resources have successfully been integrated to rice cultivars for enriching them with many features (Ali, Sanchez, Yu, Lorieux, & Eizenga, 2010; Brar & Khush, 1997; He et al., 2017; McCouch et al., 2013; Vaughan et al., 2003).

Common wild rice *Oryza rufipogon* Griff. and *O. nivara* Sharma et Shastry are the progenitors of the Asian cultivated rice and have been recognized as valuable genetic resources for rice genetic improvement (Chang, 1984; Khush, 1997; Sang & Ge, 2007; Vaughan et al., 2003). To date, many efforts have been made to investigate their classification, population genetics, evolutionary history, and adaptation potential as well as agronomical traits of the two species (Barbier, 1989; Grillo et al., 2009; Huang et al., 2012; Huang et  al., 2013; Liu, Zheng, Zhou, Zhou, & Ge, 2015; Morishima, Sano, & Oka, 1984; Zhu & Ge, 2005; Zhu, Zheng, Luo, Gaut, & Ge, 2007). Extensive studies on the population genetics of these two species have been carried out either in global scale (Banaticla‐Hilario, Berg, Hamilton, & McNally, 2013; Huang et al., 2012; Liu et al., 2015; Zhu & Ge, 2005; Zheng & Ge, 2010;) or local scale (Kuroda, Sato, Bounphanousay, Kono, & Tanaka, 2007; Pusadee, Schaal, Rerkasem, & Jamjod, 2013; Samal et al., 2018; Zhou, Xie, & Ge, 2003; Zhou et al., 2008). However, the genetic variability and population genetic structure of these two species in Sri Lanka are not well understood (Rajkumar, Weerasena, Fernando, Liyanage, & Silva, 2011). Moreover, increasing threats on the wild plant populations have been observed in Sri Lanka in the past decades due to changes in farming systems, economic development, urbanization, and other human disturbances (Rajkumar et al., 2011; Seo, Mendelsohn, & Munasinghe, 2005).

In the present study, we used microsatellite markers to evaluate the genetic variation and population genetic structure for a diverse collection of the *O. rufipogon* and *O. nivara* populations throughout the three climatic zones, that is, wet, intermediate, and dry zones, of Sri Lanka. By determining the level and patterns of genetic diversity within the two wild rice species, we expected to discover the unique genetic resources maintained in the Sri Lankan populations, to reveal their genetic relationships, and to uncover the correlations between genetic components with geographic and climatic factors. In addition, these two wild species, along with the cultivated rice have become an excellent working system for studying adaptation and speciation (Choi, Platts, Fuller, & Wing, 2017; Grillo et al., 2009; Huang et al., 2013; Liu et al., 2015; Zheng & Ge, 2010). Given the fact that Sri Lanka is an isolated island with diversified and well‐understood ecosystems (Seo et al., 2005), investigations on the Sri Lankan populations of the wild rice will contribute to a better understanding of adaptation and speciation process of the wild rice and also provide additional insights into the evolutionary history and species divergence and adaptation in general.

## 2 MATERIALS AND METHODS

### 2.1 Population sampling

**Figure 1:**
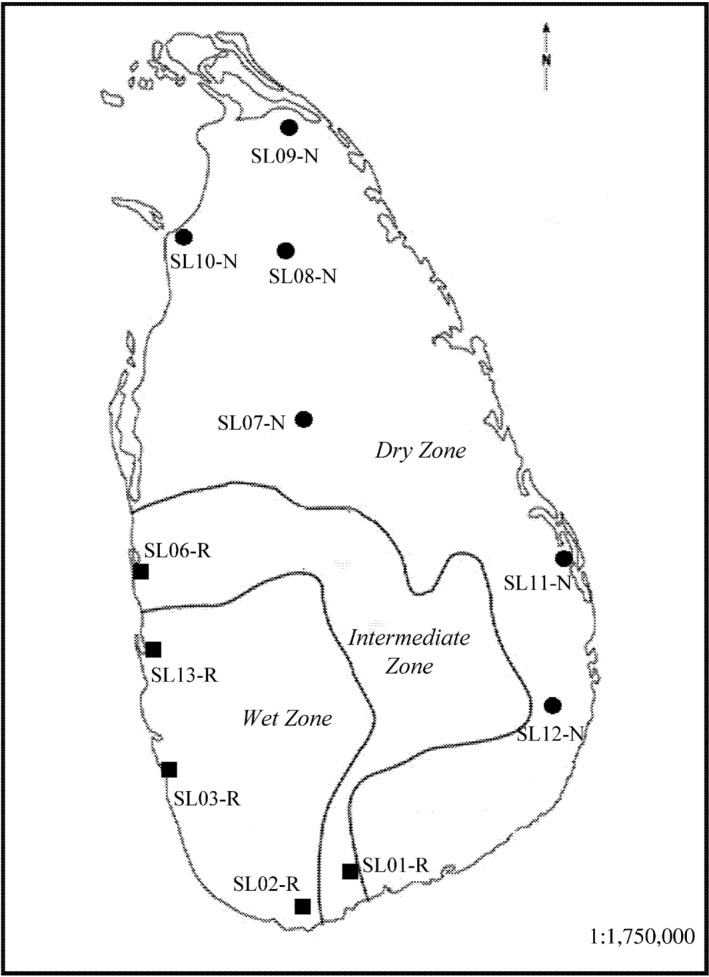
Geographical locations of 11 *Oryza rufipogon* (the squares) and *Oryza nivara* (the circles) populations sampled in this study. Dark lines separate the climatic zones in Sri Lanka. Detailed information on these populations is provided in Table S1

A total of 11 wild rice populations including five *O. rufipogon* and six *O. nivara* populations across the entire geographical distribution of the two species in Sri Lanka were collected from natural habitats (Figure 1). The sample sizes ranged from 18 to 31 individuals per population, and 132 and 183 individuals were collected for *O. rufipogon* and *O. nivara*, respectively. The green leaves were individually collected in the field and placed in zip‐lock bag containing silica gel and kept at 0–4°C until DNA extraction. To prevent repeated samples of same genets (clones), the distance between sampled individuals was at least 5 m apart. The detailed information of these populations is provided in Supporting Information Table S1.

### 2.2 DNA extraction and PCR amplification

The gDNA was extracted from 20 mg of silica gel‐dried leaves, using the Plant genomic DNA kit (Biomed DL114‐01) following by the CTAB protocol (Saghai‐Maroof, Soliman, Jorgensen, & Allard, 1984) and further quantified using a Nanodrop 2000 (Scientific, 2009). Thirty‐three microsatellite (Simple Sequence Repeat – SSR) primer pairs designed from cultivated rice were used to assay genetic variation of all included materials, based on the RiceGenes Database (https://gramene.org). Detailed information of the primer pairs is given in Table S2. Primers were synthesized by ABI (Applied Biosystems, Foster City, CA, USA), with the forward primers labeled with blue (FAM), yellow (TAMAR), and green (HEX) fluorophores, respectively. The DNA amplification was carried out using a 2,720 thermal cycler (Applied Biosystems) in a 15 µl reaction mixture. Each reaction contained 5 µg buffer (ddH_2_O), 7 µg 2× Taq PCR MasterMix (0.1 U Taq Polymerase/μl, 500 μM dNTP each, 20 mM Tris–HCl, 100 mM KCl, 3 mM MgCl2), 1 µl each of the forward and reverse primer (10 µM), and 1 µg template DNA. The PCR cycle profile was 94°C, 3 min; 35 cycles of 94°C, 30 s, 55°C, 30 s, and 72°C, 1 min; and 72°C, 10 min for the final extension.

The PCR products (2 µl) were diluted in 8 µl of ultrapure water and scoured in 100% alcohol for 15 min. The diluted DNA was dried and mixed with highly deionized formamide (Applied Biosystems) and then submitted for fragment analysis by capillary electrophoresis using an Applied Biosystems 3130×l DNA analyzer (Applied Biosystems). The DNA fragment size analysis and allele calling were performed using GeneScan and GeneMapper software (Applied Biosystems), followed by the manual allele binning. The matrix standard kit was used to generate the “multicomponent matrix” when analyzing the FAM‐, TAMRA‐, and HEX‐labeled DNA fragments with the ABI3130 series systems. Data Collection Software (Applied Biosystems) used the multi component matrix for the instrument to analyze automatically for three different colored fluorescent dye‐labeled samples in a single capillary. In addition, reference lanes were further verified by polyacrylamide gel electrophoresis. In the case of non‐amplification, the PCR was repeated to exclude technical failure and a null allele was recorded if both PCRs failed.

### 2.3 Data analysis

All SSR loci were initially evaluated for the adherence to the Hardy–Weinberg equilibrium and for presence of null alleles using the method based on heterozygous deficiency (Brookfield, 1996). Genetic parameters for each population were assessed by calculating the allelic richness (*A*), the mean observed heterozygosity (*H*_O_), the percentage of polymorphic loci (*P*), and the unbiased expected heterozygosity (*H*_E_). All the estimates of genetic diversity were calculated using GenAIEx 6.5.01 software (Peakall & Smouse, 2006).

To identify the number of distinct clusters that best explain the genetic structure of the data set, we performed the structure analysis using the program STRUCTURE 2.3.4 (Falush, Stephens, & Pritchard, 2003; Pritchard, Stephens, & Donnelly, 2000), with default parameters. The *K* values ranging from 1 to 12 were evaluated using 12 independent runs for each *K* and 10^6^ MCMC steps, assuming a burn‐in period of 10^4^ steps. The suggested *K* values were evaluated using the ∆*K* statistic developed by Evanno, Regnaut, and Goudet (2005). The level of genetic structure among the *K* clusters suggested by this analysis was evaluated. We also assessed both inter‐ and intra‐specific genetic structure of the populations *via* multivariate principal component analyses (PCoA) through the dudi.pca() function of “adegenet” R package (Jombart & Ahmed, 2011). An analysis of molecular variance (AMOVA) were performed, implemented in GenAIEx 6.5.01 software (Peakall & Smouse, 2006), to assess the proportion of genetic variance explained by the differences within and between populations. An UPGMA clustering analysis of all populations was performed based on Nei's (1972) unbiased genetic distance, and the tree was visualized with TREEVIEW ver. 1.52 (Page, 1996). We also calculated the *F*_ST_ value using FSTAT version 2.9.3 (Goudet, 2002) to investigate the group relationship.

### 2.4 Species distribution modeling

A species distribution model (SDM) was generated for *O. rufopogon* and *O. nivara* using the software MAXENT 3.3.3 (Phillips & Dudík, 2008; Phillips, Anderson, & Schapire, 2006) based on the maximum entropy model of geographical distribution of species. This method combines existing data with ecological climate layers to predict the relative probability of the existence of the species in a certain geographical area (Phillips et al., 2006). The occurrence of *O. rufopogon* and *O. nivara* were recorded from the Gateway to Genetic Resources (https://www.genesys-pgr.org/). To estimate the relative contributions of the environmental variables on the species distribution, we performed multiple relative importance tests including the percentage contribution, permutation test, and jackknife test. To predict species occurrence, environmental layers of 19 biologically meaningful climate variables (BIO1‐19) were downloaded from the WorldClim database (https://www.worldclim.org/; Hijmans, Cameron, Parra, Jones, & Jarvis, 2005) under present day (1950–2000), the last glacial maximum (LGM; ~21kya) and the last interglacial (LIG; ~120 to 140kya) conditions. In addition, we added 107 *O. nivara* and 38 *O. rufipogon* records collected from Sri Lanka from field investigations. We defined the study extent according to the commonly accepted range of the two species, between 5.55^0^N and 9.51^O^N, 79.42^O^E and 81.53^O^E**.** All the 145 localities of our own collection sites were used to build the models using the default parameters for convergence threshold (10^–5^) and number of iterations (500). To insure the consistency of the model predictions, 75% of the localities were used to train the model and 25% were used to test it.

## 3 RESULTS

Based on the preliminary screening of 150 SSR primer pairs across representative individuals sampled from different populations, the 33 SSRs produced clear and reproducible products and thus, chosen for genotyping the populations used in this study. The 33 loci were dispersed in all 12 chromosomes in cultivated rice with the polymorphism varied widely among loci (Table S2). The genotypic linkage disequilibrium among the loci did not show significant values (*p* > 0.05), suggestive of an absence of linkage disequilibrium between loci. All 33 loci displayed polymorphism among the 11 populations with a total of 172 alleles identified. The most variable locus (RM426) had 30 alleles, while the RM457 showed only three alleles across the populations, with an average of 11.66 alleles per population (Table S2). The table of allelic frequencies of each population is available from the first author upon request.

We calculated the genetic diversity at both population and species levels and found a moderate to high genetic diversity (Table 1). At population level, the genetic diversity varied among 11 populations, with the average of *A *= 2.05, *p *= 81.82, and *H*_E_ **= 0.369. As measured by allelic richness and expected heterozygosity, population SL13‐R and population SL11‐N maintained the highest (*A *= 2.47, *H*_E_ **= 0.481) and the lowest (*A *= 1.72, *H*_E_ **= 0.294) diversity, respectively. It is evident that *O. rufipogon* populations maintain higher level of genetic diversity than those of *O. nivara* populations (Table 1).

**Table 1:** Genetic parameters characterizing 11 *O. rufipogon* and *O. nivara* populations in Sri Lanka

Population code	*N*	*A*	*P*	*H*_O_	*H*_E_
*O. rufipogon*					
SL01‐R	18	2.04	90.91	0.683	0.418
SL02‐R	31	2.17	93.94	0.701	0.451
SL03‐R	31	2.08	75.76	0.443	0.375
SL06‐R	25	1.75	72.73	0.566	0.333
SL13‐R	27	2.47	90.91	0.524	0.481
Average	26.4	2.10	84.85	0.583	0.411
*O*.* nivara*					
SL07‐N	32	1.99	81.82	0.335	0.322
SL08‐N	32	2.05	63.64	0.374	0.329
SL09‐N	31	2.43	100.0	0.358	0.426
SL10‐N	29	1.90	84.85	0.324	0.316
SL11‐N	29	1.72	60.61	0.268	0.294
SL12‐N	30	1.92	84.85	0.266	0.315
Average	30.5	1.96	79.29	0.320	0.333
Total average	27.2	2.05	81.82	0.440	0.369

Note

*A*: Average number of alleles; *H*_E_ and *H*_O_: expected and observed heterozygosity, respectively; *N*: average number of samples per population; *P:* percentage of polymorphic loci.

**Figure 2:**
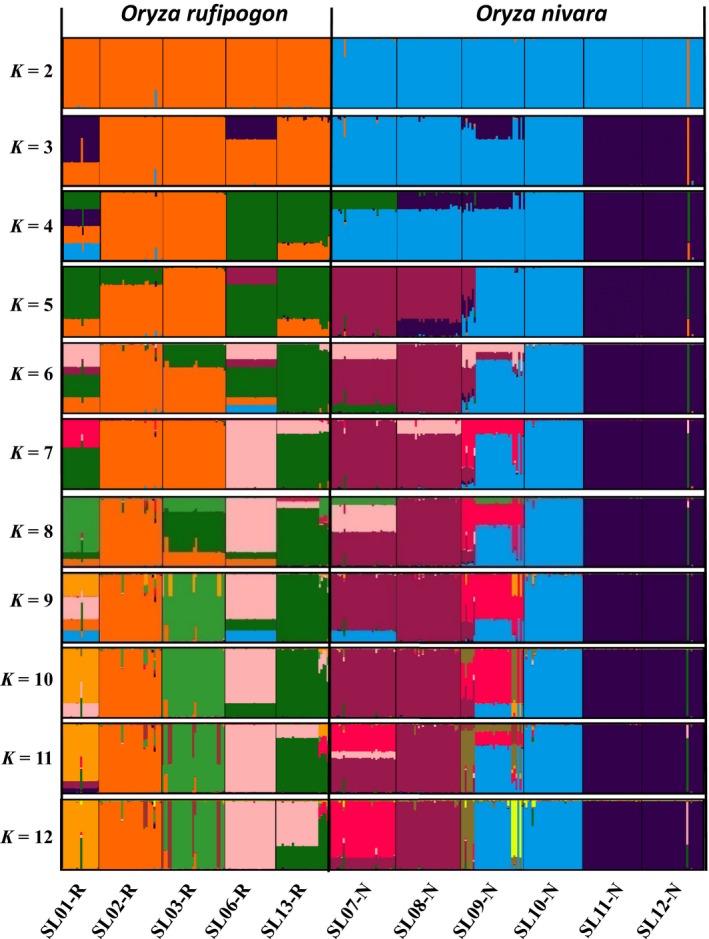
STRUCTURE results by model‐based population assignment at *K* from 2 to 6. Each vertical bar represents an individual, with its assignment probability to genetic clusters represented by different colors

**Figure 3:**
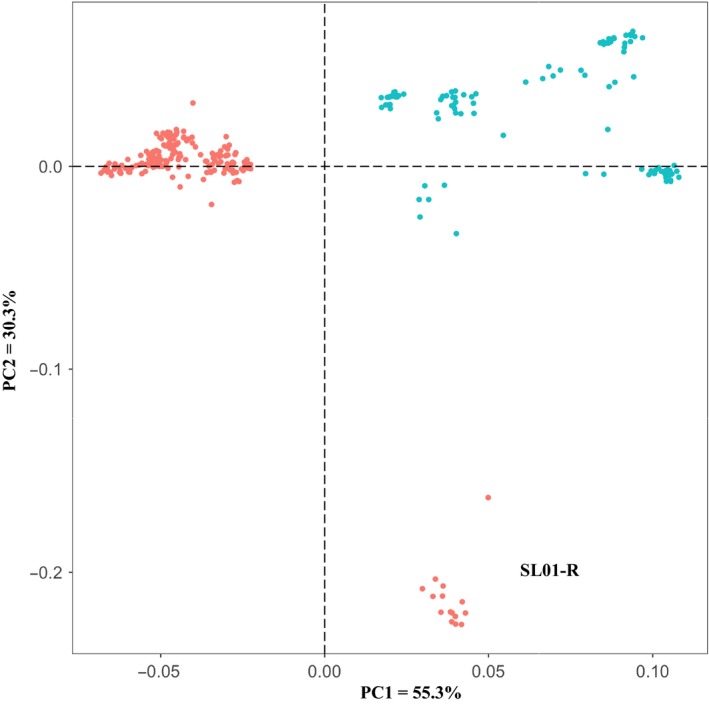
Principal component analysis (PCoA) showing the clustering of genotypes at 33 microsatellite loci, with all individuals scored for PC1 versus PC2. The colors representing different species are the same as for Figure 2

**Figure 4:**
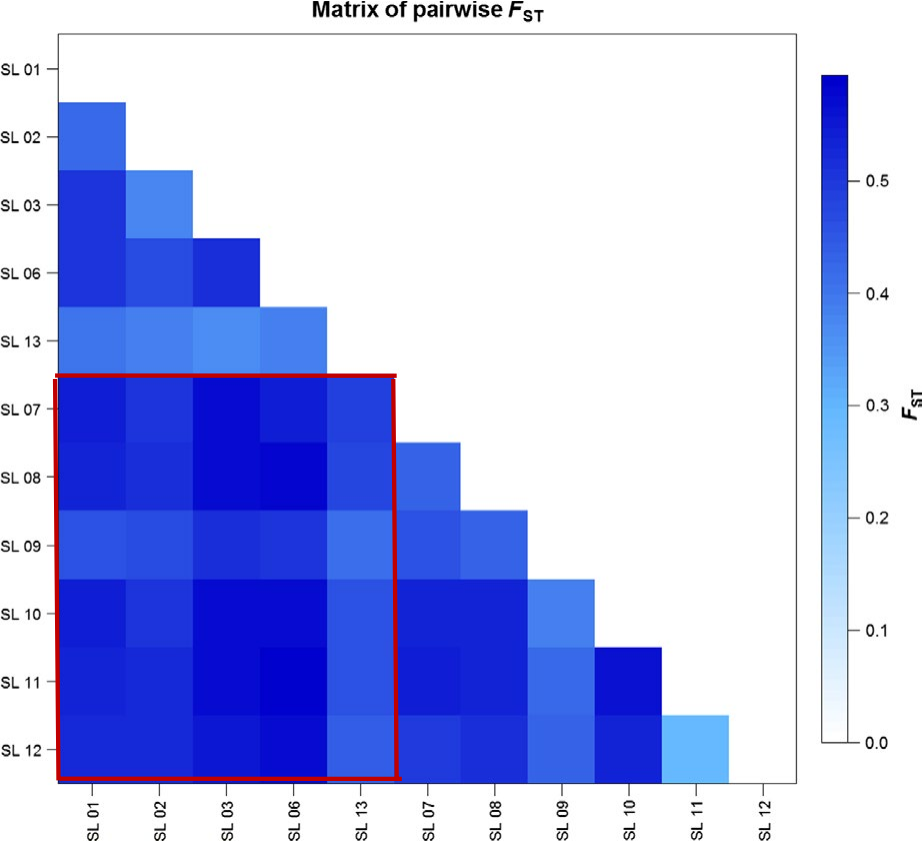
Matrix of pairwise *F*_ST_ values of all 11 populations. The box in red indicates the among‐population values between species

**Figure 5:**
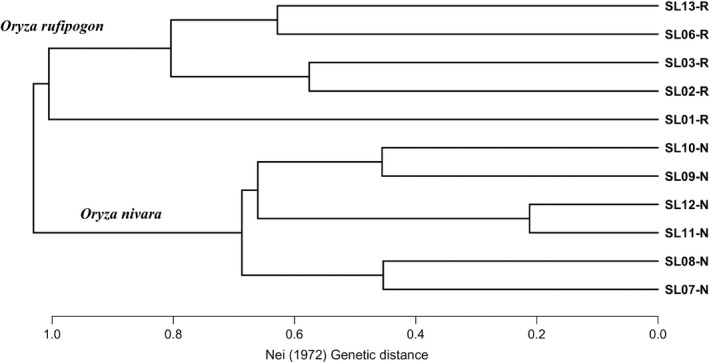
The UPGMA cladogram based on Nei (1972) genetic distance. The population codes are the same as for Figure 1

The STRUCTURE analysis done by running the *K* values from 2 to 12 based on the allele frequency distribution showed that the first divergence (*K *= 2) occurred between two major groups (i.e., two species; Figure 2). From *K *= 3 to *K *= 12, each species was further divided into distinct subgroups, corresponding to the populations within species. It is evident that there is admixed genetic background for individuals in some populations although a majority of individuals could be clearly assigned to a single population. Interestingly, the *O. rufipogon* populations with many admixed individuals (SL01‐R and SL06‐R) were sampled from the intermediate zone (Figure 1). The PCoA also confirms the two major groups, with SL01‐R being in a different place in the plot, a reflection of its admixture feature (Figure 3). The AMOVA showed that a large proportion of the total genetic diversity existed within (47.7%) and among populations (40%), with the genetic differentiation between species relatively low (12.3%) but significant (Table 2). For specific species, 43.7% and 48.5% of the total variation were partitioned among the populations for *O. rufipogon* and *O. nivara*, respectively. The high genetic differentiation was reflected by the high *F*_ST_ values (Figure 4). The UPGMA tree illustrated similar results, *that is,* all 11 populations were genetically structured into two well‐separated major groups (species) and further divided to populations (Figure 5).

**Table 2:** AMOVA analysis of 11 *O. rufipogon* and *O. nivara* populations sampled in this study

Group	*df*	SS	V	%
Combination of *O. rufipogon* and *O. nivara*				
Between species	1	737.983	1.48515	12.31***
Among populations	9	2,535.578	4.82904	40.02***
Within populations	619	3,559.825	5.75093	47.67***
*O. rufipogon*				
Among populations	4	1,046.302	4.87539	43.69***
Within populations	259	1627.183	6.28256	56.31***
*O. nivara*				
Among populations	5	1,440.382	4.64331	48.50***
Within populations	360	1775.189	4.93108	51.50***

Note

SS: Sum of squares; V: Variance components; %: Percentage of variation.

***p < 0.001.

**Figure 6:**
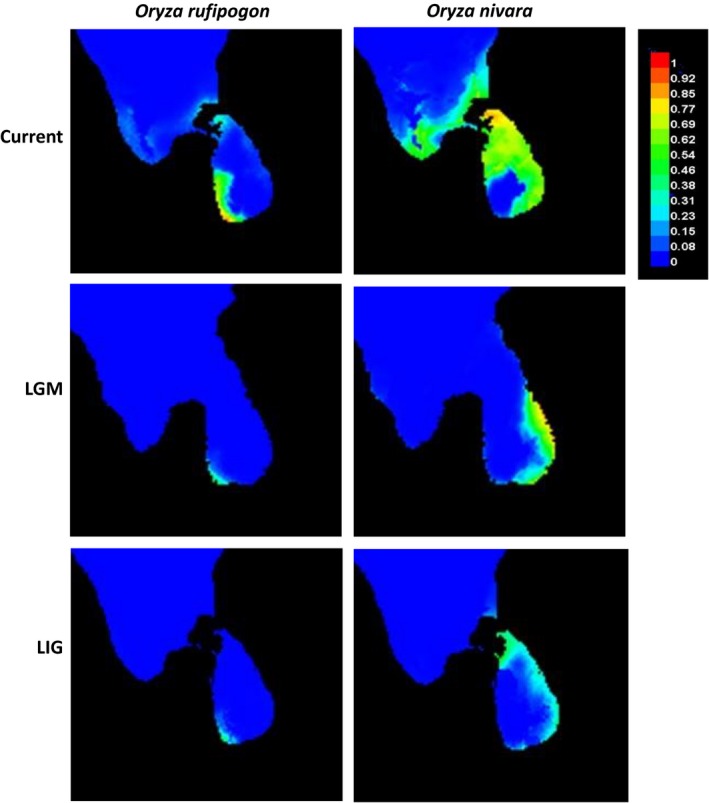
Average species distribution of *Oryza rufipogon* and *Oryza nivara* predicted by SDM based on 19 bioclimatic variables representing the current, LGM (~21 kya), and LIG (120–140 kya) climatic conditions, respectively. Warmer colors show areas with a higher probability of presence

Finally, the SDMs under present, LGM and LIG with all 19 bioclimatic variables showed that the estimated current distribution patterns of the two species were highly consistent with their actual distribution areas at present in Sri Lanka (Figure 6), *that is*,* O. nivara* occurred widely in the dry zone while *O. rufipogon* confined to wet zone. It is interesting to note that the predicted distribution areas at the LGM and LIG were much smaller than at present for both species, indicating a significant range expansion of the two species in Sri Lanka since the last interglacial around 120–140 kya. The results of the multiple relative importance tests indicated that annual mean temperature (BIO1) is the most important variable for determining the distribution of both species. In addition, the mean temperature of coldest quarter (BIO11) and the mean diurnal range (BIO2) were important for the distribution of *O. rufipogon*, while the mean temperature of driest quarter (BIO9) and the mean temperature of wettest quarter (BIO8) were critical for the distribution of *O. nivara*, indicating the differences in habitat preference of the two species.

## 4 DISCUSSION

Microsatellites (SSRs) are featured as co‐dominant, highly polymorphic, abundant, and randomly distributed markers in the genomes and have been successfully used for a variety of studies in rice biology (Kuroda et al., 2007; McCouch et al., 1997; Pusadee et al., 2013; Samal et al., 2018; Wang et al., 2014; Zhou et al., 2003). As SSR markers have good cross‐species amplification to the close relatives, they provide a powerful tool for studying *O. nivara* and *O. rufipogon* given that thousands of SSR markers across rice genome have been well characterized (Cho et al., 2000; McCouch et al., 1997).

### 4.1 Genetic diversity and population genetic structure of *O. rufipogon* and *O. nivara* in Sri Lanka

To date, many investigations on genetic diversity of *O. rufipogon* and *O. nivara* have been undertaken using SSR markers and relatively high genetic diversity has been detected at both population and species levels (Banaticla‐Hilario et al., 2013; Kuroda et al., 2007; Pusadee et al., 2013; Samal et al., 2018; Zhou et al., 2003;). In a study on the Chinese *O. rufipogon* populations, Zhou, Wang, Davy, and Liu (2013) found a genetic diversity of 0.413 and 0.787, as measured by *H*_E_, at population and species levels, respectively. Kuroda et al. (2007) studied the wild populations of both species in the Vientiane Plain of Laos and obtained the *H*_E_ values of 0.37–0.67 for *O. rufipogon* and 0–0.44 for *O. nivara* at the population level and 0.77 for *O. rufipogon* and 0.64 for *O. nivara* at the species level. Using 29 SSR markers, Banaticla‐Hilario et al. (2013) investigated 119 accessions of three wild rice species (*O. rufipogon*,* O. nivara,* and *O. meridionalis*) across Asia Pacific and found that the average *H*_E_ values for the species was 0.70 and 0.67 for *O. rufipogon* and *O. nivara*, respectively, with the lowest for the Nepalese *O. nivara* (0.39) and the highest for the Southeast Asian *O. rufipogon* (0.66). In a recent study on 418 *O. rufipogon* and *O. nivara* accessions collected across the entire Indian peninsula, Singh et al. (2018) reported that the genetic diversity was *H*_E_ **= 0.63 (*O. rufipogon*) and 0.64 (*O. nivara*), respectively.

The present study yielded the first report on the population genetics of the natural populations of the two wild rice species in Sri Lanka. Although the number of the sampled populations is not large, the results based on the representative populations collected across the entire Sri Lanka provide useful information on the populations of the two species. Our estimates of the genetic diversity of the two species in Sri Lanka, either at population level (0.41 for *O. rufipogon* and 0.33 for *O. nivara*) or at the species level (0.62 for *O. rufipogon* and 0.58 for *O. nivara*) were relatively high given the small area of the island. This result indicates that the wild rice populations in Sri Lank may serve as an important genetic reservoir for rice improvement and deserve effective conservation. Consistent with previous studies (Liu et al., 2015; Pusadee, Jamjod, Rerkasem, & Schaal, 2016; Zheng & Ge, 2010), we detected a significantly but low level of divergence between species (Table 2), suggesting that these two species is still in the process of speciation with potential gene flow between them. This speculation is supported by the presence of admixture populations (SL01‐R and SL06‐R) in the intermediate climatic zone.

It is well recognized that the perennial *O. rufipogon* is predominantly outcrossing while the annual *O. nivara* is highly self‐fertilizing (Sang & Ge, 2007). Therefore, the *O. rufipogon* populations exhibited significantly lower differentiation than the *O. nivara* populations, as detected by different molecular markers in previous studies (Kuroda et al., 2007; Liu et al., 2015; Pusadee et al., 2016). In contrary to the previous studies, we found that the *O. rufipogon* populations have comparable levels of population differentiation relative to the *O. nivara* populations in Sri Lanka, with the among‐population diversity being 43.7% for *O. rufipogon* and 48.5% for *O. nivara* (Table 2). Such a unique pattern implies a low level of gene flow among *O. rufipogon* populations, which might result from the isolated populations in the wet zone of Sri Lanka and need further investigation by dense sampling in this area.

### 4.2 Evolutionary history and origin of *O. nivara* in Sri Lanka

The hypothesis that *O. nivara* originated from *O. rufipogon* to associate with an ecological shift from a persistently wet to a seasonally dry habitat (Barbier, 1989; Vaughan, Lu, & Tomooka, 2008) has been supported by many recent studies (Grillo et al., 2009; Liu et al., 2015; Zheng & Ge, 2010). Interestingly, these two species were sympatric in many areas across their entire geographic distribution (Liu et al., 2015; Vaughan et al., 2008), which raised an interesting question that where and how the derived *O. nivara* originated to adapt to new environments. Zheng and Ge (2010) estimated that *O. nivara* was derived from the *O. rufipogon* populations about 0.16 million years ago around LIG to associate with an ecological shift from a persistently wet to a seasonally dry habitat. Based on the sequences of 12 nuclear and chloroplast loci, Liu et al. (2015) further studied 26 wild populations across the entire geographic ranges of the two species and hypothesized that *O. nivara* might have independently originated multiple times from different *O. rufipogon* populations, with Sri Lanka as one of the potential areas of origin. However, the climate variables that have contributed to the origin of *O. nivara* remain plausible. In the present study, we used SDM to detect the dynamics of the preferred habitats for the two species and demonstrated that both *O. rufipogon* and *O. nivara* populations have expanded substantially since the last internal glacial, particularly for *O. nivara* (Figure 6). In addition, we identified a few of the key environmental variables that have contributed to the distribution of the two species.

One interesting and challenging question is whether the Sri Lankan *O. nivara* originated locally within the island or introduced from the areas outside the island. Pant and Kumar (1997) reported that similar climatic conditions arose between the plains of southern India and northern Sri Lanka since the late Pleistocene. Evidence also showed that Sri Lanka has been connected with Indian subcontinent and the dispersal across the Palk Strait between southeastern India and northern Sri Lanka was possible during the LGM (21 kya; Figure 6). In addition, the well‐understood climatic zones in Sri Lanka and their close match to the distribution of the two species (*i.e*., *O. rufipogon* in the wet zone and *O. nivara* in the dry zone) provide a unique system to investigate adaptive divergence and ecological speciation in plant species. Together, our study on *O*. *nivara* and *O. rufipogon* populations in Sri Lanka not only provide important insights into the population genetics and evolution history of these wild species, but also is of great significance for in situ conservation and management of these genetic resources.

## CONFLICT OF INTEREST

None declared.

## AUTHOR CONTRIBUTIONS

S. G., D. R., and S. S. conceived the ideas. D. R. and Q‐L. M. collected the data. S. S., A. T., and A. M. analyzed the data; and S. G., A. T., A. M., B. M., and S. S. wrote the paper.

## DATA ACCESSIBILITY

Data available from the Dryad Digital Repository.
