# Supplementary material for: Population genetics and evolutionary history of the wild rice species Oryza rufipogon and O. nivara in Sri Lanka
Source: Ecol Evol. 2018 Nov 11;8(23):12056–65. doi: 10.1002/ece3.4665 (PMC6303766; doi:10.1002/ece3.4665)
Supplement: Supplementary file 1 [file ECE3-8-12056-s001.docx]

**Supplementary Tables**

**Table S1.** Information on the 11 wild rice populations sampled in this study.

| **Population**  **Code** | **Location** | **Latitude** | **Longitude** | **Elevation (m)** | **Sample size** |
| --- | --- | --- | --- | --- | --- |
| ***O. rufipogon*** | | | | | |
| SL01-R | Weerakatiya, Hambantota | 06°09’18.6” | 80°46’02.8” | 62 | 18 |
| SL02-R | Thihagoda,  Matara | 06°00’01.6” | 80°33’43.9” | 03 | 31 |
| SL03-R | NugagodaJunction, Waskaduwa Kalutara | 6°38’ 07.1” | 79°57’03.2” | 30 | 31 |
| SL06-R | Madampe,  Puttalam | 07°28’02.9” | 79°49’41.0” | 05 | 25 |
| SL13-R | Colombo-Negambo Rd, Seeduwa | 07°06' 51.4” | 79°53' 15.3” | 09 | 27 |
| ***O. nivara*** | | | | | |
| SL07-N | Maradankadawala, Anuradhapura | 08°07’44.9” | 80°33’46.9” | 132 | 32 |
| SL08-N | 105 km post, A-9 Rd, Vavuniya | 08°51’06.0” | 80°29’06.1” | 90 | 32 |
| SL09-N | Paranthan-Mullaitive Rd, Kilinochchi | 09°25’55.2” | 80°29’56.5” | 08 | 31 |
| SL10-N | Sirnavetkulum, Mannar Rd, Manna | 08°54’36.4” | 79°57’42.3” | 06 | 29 |
| SL11-N | Vellavelly,Batticaloa | 07°30’33.1” | 81°43’55.1” | 06 | 29 |
| SL12-N | Siyabalnduwa-  Lahugala Rd, Badulla | 06°53’48.8” | 81°41’22.9” | 35 | 30 |

**Table S2. SSR primer pairs used for DNA amplification in this study**

| **Locus** | **Chromosome Location** | **Repeat Type and Length** | **Forward Primer** | **Reverse Primer** | **Average No. Alleles** |
| --- | --- | --- | --- | --- | --- |
| RM119 | 4 | (GTC)6 | CATCCCCCTGCTGCTGCTGCTG | CGCCGGATGTGTGGGACTAGCG | 5 |
| RM129 | 1 | (CGG)8 | TCTCTCCGGAGCCAAGGCGAGG | CGAGCCACGACGCGATGTACCC | 8 |
| RM164 | 5 | (GT)16TT(GT)4 | TCTTGCCCGTCACTGCAGATATCC | GCAGCCCTAATGCTACAATTCTTC | 19 |
| RM167 | 11 | (GA)16 | GATCCAGCGTGAGGAACACGT | AGTCCGACCACAAGGTGCGTTGTC | 17 |
| RM18 | 7 | (GA)4AA(GA)(AG)16 | TTCCCTCTCATGAGCTCCAT | GAGTGCCTGGCGCTGTAC | 14 |
| RM184 | 10 | (CA)7 | ATCCCATTCGCCAAAACCGGCC | TGACACTTGGAGAGCGGTGTGG | 5 |
| RM190 | 6 | (CT)11 | CTTTGTCTATCTCAAGACAC | TTGCAGATGTTCTTCCTGATG | 7 |
| RM234 | 7 | (CT)25 | ACAGTATCCAAGGCCCTGG | CACGTGAGACAAAGACGGAG | 14 |
| RM247 | 12 | (CT)16 | TAGTGCCGATCGATGTAACG | CATATGGTTTTGACAAAGCG | 11 |
| RM263 | 2 | (CT)34 | CCCAGGCTAGCTCATGAACC | GCTACGTTTGAGCTACCACG | 13 |
| RM267 | 5 | (GA)21 | TGCAGACATAGAGAAGGAAGTG | AGCAACAGCACAACTTGATG | 11 |
| RM273 | 4 | (GA)11 | GAAGCCGTCGTGAAGTTACC | GTTTCCTACCTGATCGCGAC | 11 |
| RM293 | 3 | (GT)20 | TCGTTGGGAGGTATGGTACC | CTTTATCTGATCCTTGGGAAGG | 5 |
| RM321 | 9 | (CAT)5 | CCAACACTGCCACTCTGTTC | GAGGATGGACACCTTGATCG | 5 |
| RM336 | 7 | (CTT)18 | CTTACAGAGAAACGGCATCG | GCTGGTTTGTTTCAGGTTCG | 21 |
| RM404 | 8 | (GA)33 | CCAATCATTAACCCCTGAGC | GCCTTCATGCTTCAGAAGAC | 20 |
| RM409 | 9 | (AGC)8 | CCGTCTCTTGCTAGGGATTC | GGGGTGTTTTGCTTTCTCTG | 8 |
| RM414 | 1 | (TGCA)6 | ATTGCAGTCATGCAGCAGTC | ATATCTCCAATGTGGCAGGG | 6 |
| RM425 | 2 | (CGG)9 | CCAACGAAGATTCGAAGCTC | CAGCACCATGAAGTCGCC | 12 |
| RM426 | 3 | (CA)10 | ATGAGATGAGTTCAAGGCCC | AACTCTGTACCTCCATCGCC | 30 |
| RM457 | 11 | (TTAA)5 | CTCCAGCATGGCCTTTCTAC | ACCTGATGGTCAAAGATGGG | 3 |
| RM474 | 10 | (AT)13 | AAGATGTACGGGTGGCATTC | TATGAGCTGGTGAGCAATGG | 18 |
| RM477 | 8 | (AATT)5 | TCTCGCGGTATAGTTTGTGC | ACCACTACCAGCAGCCTCTG | 7 |
| RM490 | 1 | (CT)13 | ATCTGCACACTGCAAACACC | AGCAAGCAGTGCTTTCAGAG | 10 |
| RM505 | 7 | (CT)12 | AGAGTTATGAGCCGGGTGTG | GATTTGGCGATCTTAGCAGC | 8 |
| RM507 | 5 | (AAGA)7 | CTTAAGCTCCAGCCGAAATG | CTCACCCTCATCATCGCC | 9 |
| RM511 | 12 | OSJNBA0093A14 | CTTCGATCCGGTGACGAC | AACGAAAGCGAAGCTGTCTC | 6 |
| RM515 | 8 | (GA)11 | TAGGACGACCAAAGGGTGAG | TGGCCTGCTCTCTCTCTCTC | 13 |
| RM545 | 3 | (GA)30 | CAATGGCAGAGACCCAAAAG | CTGGCATGTAACGACAGTGG | 15 |
| RM566 | 9 | (AG)15 | ACCCAACTACGATCAGCTCG | CTCCAGGAACACGCTCTTTC | 23 |
| RM567 | 4 | (GA)21 | ATCAGGGAAATCCTGAAGGG | GGAAGGAGCAATCACCACTG | 18 |
| RM71 | 2 | (ATT)10T(ATT)4 | CTAGAGGCGAAAACGAGATG | GGGTGGGCGAGGTAATAATG | 5 |
| RM7102 | 12 |  | TAGGAGTGTTTAGAGTGCCA | TCGGTTTGCTTATACATCAG | 8 |
